# Association of polymorphisms in *APOE*, *p53*, and *p21* with primary open-angle glaucoma in Turkish patients

**Published:** 2009-06-30

**Authors:** E. Saglar, D. Yucel, B. Bozkurt, R.K. Ozgul, M. Irkec, A. Ogus

**Affiliations:** 1 Hacettepe University, Faculty of Science, Department of Molecular Biology, Ankara, Turkey; 2 Selcuk University, Meram Medical Faculty, Department of Ophthalmology, Konya, Turkey; 3 Hacettepe University, Institute of Child Health, Department of Pediatrics, Ankara, Turkey; 4 Hacettepe University, Faculty of Medicine, Department of Ophthalmology, Ankara, Turkey

## Abstract

**Purpose:**

To investigate the association between Apolipoprotein E (*APOE*), tumor suppressor protein p53 (*p53*), and **c**yclin-dependent kinase inhibitor 1A (*p21*) genes and primary open-angle glaucoma (POAG) in a cohort of Turkish subjects.

**Methods:**

Seventy-five POAG patients (49 women, 26 men) and 119 healthy subjects (67 women, 52 men) were genotyped with polymerase chain reaction-restriction fragment length polymorphism (PCR-RFLP). Allele and genotype frequencies between healthy subjects and glaucoma patients were compared by the χ^2^ test, and intraocular pressure (IOP), cup/disc ratio (C/D) and visual field indices (MD and PSD) were compared among different *APOE*, *p53*, and *p21* genotypes in POAG group. A p value <0.05 was considered as statistically significant.

**Results:**

The mean ages were 63.8±9.5 and 61.8±10.2 years in POAG and control groups, respectively (p=0.18). There were no significant differences in the distribution of *APOE, p53*, and *p21* genotypes between the healthy subjects and POAG patients (p=0.38, p=0.12, and p=0.2, respectively). There were no significant differences in maximum IOP, MD, and PSD values among different groups of *p53* and *p21* genotypes (p>0.05). POAG subjects with the ε2ε3 genotype had a worse PSD value (median=2.2) than those with the ε3ε4 genotype (median=1.77; p=0.01) and POAG subjects with the ε3ε3 genotype had worse MD and PSD values (median= -7.4 and 3.4, respectively) than those with the ε3ε4 genotype (median= -4.1 and 1.77, respectively; p=0.034 and 0.028, respectively).

**Conclusions:**

Our study found no link between polymorphisms in *APOE, p53*, and *p21* genes and POAG in Turkish patients, although a larger sample is required to elucidate the role of these polymorphisms in the pathogenesis and course of glaucoma.

## Introduction

Glaucoma is a degenerative optic neuropathy, characterized by optic nerve head (ONH) changes and visual field loss. Elevated intraocular pressure (IOP) is generally accepted as the major modifiable risk factor for glaucoma, however, factors other than IOP also play role in the pathogenesis and progression of glaucoma, particularly in subjects with normal tension glaucoma (NTG). It is the second leading cause of blindness worldwide, estimated to affect about 70 million people, with 6.7 million of these being bilaterally blind [1]. Primary open-angle glaucoma (POAG, OMIM 137760) is the major type of primary glaucoma in most populations. POAG is a genetically heterogeneous disorder and at least 22 genetic loci have been mapped for POAG of which only *GLC1A* (myocilin*, MYOC*), *GLC1E* (optineurin, *OPTN*), *GLC1G* (WD repeat domain 36, *WDR36*), and *GLC3A* (*cytochrome P4501B1, CYP1B1*) have been characterized [2-7]. However, mutations in these genes account for less than 10% of POAG cases. It appears that POAG is a complex trait and multiple genes, each with allelic variations, and environmental factors contribute to the pathogenesis and phenotype and increase individual’s susceptibility to glaucomatous optic neuropathy, with no particular gene having a single dominant effect.

Apolipoprotein E (*APOE*), which is the major apolipoprotein in the central nervous system, plays an important role in neural function and repair after injury. APOE is up-regulated in response to oxidative stress and is endowed with antioxidant properties [8]. It exists as three common isoforms E2, E3 and E4, encoded by different alleles (ε2, ε3, and ε4) on a single gene on chromosome 19 (OMIM 107741). Possession of the ε4 allele was shown to be associated with a reduced outcome after traumatic head injury [9,10] and increased risk of earlier development of Alzheimer’s disease [11,12]. In the rat eye, it has been shown to be synthesized by Müller cells, secreted in the vitreous, absorbed by the retinal ganglion cells (RGC), and transported down the optic nerve [13]. Its possible role in RGC metabolism, together with its documented effect on neuronal survival following ischemic and traumatic insults, has led to the hypothesis that particular APOE isoforms could be related to neuronal damage in glaucoma patients [14]. Given the potential similarities between the cellular events leading to degeneration in both Alzheimer’s disease and glaucoma, the higher incidence of glaucoma in Alzheimer’s disease [15,16] and APOE ε4 allele as a risk factor for Alzheimer’s disease, APOE seems to be a pliable candidate for glaucoma susceptibility. In the literature, some of the studies showed an association between certain types of APOE alleles and glaucoma [14,17-21], whereas others found no association [22-25].

Apoptosis is a form of genetically controlled, programmed cell death and an important mechanism responsible for RGC death in glaucoma [26,27]. One of the primary regulatory steps of apoptosis is the activation of the tumor suppressor protein, p53, which is encoded by the *TP53* gene (guardian of the cell) located on the short arm of chromosome 17 (17p13.1) in humans. p53 functions as a transcription factor that can upregulate the expression of the pro-apoptotic gene *bax* and downregulate the expression of the antiapoptotic gene *bcl-2*. This protein has been described as "the guardian of the genome," "the guardian angel gene," and the "master watchman," referring to its role in conserving stability by preventing genome mutation [28]. Mutations of *p53* have been detected in almost 50% of human malignancies, since the mutant or inactivated p53 protein fails to initiate the apoptotic process and, consequently, genetically damaged cells proliferate in an uncontrolled manner [29]. In neurodegenerative processes and toxic neuronal injury, *p53* is upregulated, thereby promoting cell death through apoptosis [30]. Genetic polymorphic variants of *p53* were shown to be associated with POAG [31,32].

Cyclin-dependent kinase (CdK) inhibitor 1A (p21, Cip1), is a protein which in humans is encoded by the *CDKN1A* gene located on chromosome 6. It is known to cause irreversible G_1_ arrest in human fibroblasts by mediating the inhibition of CdKs [33]. *p21* is the major transcriptional target of p53; despite this, loss-of-function mutations in *p21* (unlike *p53*) do not predispose to cancer incidence. A single nucleotide polymorphism (SNP) in *p21* which consists of a C to A transverse change at the third base of codon 31, resulting in the exchange of a Serine for an Arginine, has been reported [34]. This codon 31 polymorphism is thought to encode a DNA-binding zinc-finger domain [35]. A study on a Chinese population showed an association between the Arg form of the *p21* codon 31 polymorphism and POAG, suggesting that this allele may alter the state of apoptosis in glaucomatous optic neuropathy, failing to protect the ganglion cells [36].

In the present study, we evaluated the genetic association of *APOE*, *p53*, and *p21* polymorphisms with POAG in a group of Turkish subjects and investigated their possible involvement with the disease phenotype and severity.

## Methods

The cases enrolled in this study were unrelated to each other and were derived exclusively from the Turkish population. All subjects have undergone systematic examination of optic disc, visual field examination with automated static white on white threshold perimetry using the 30-2/24-2 program of the Humphrey Field Analyzer (Dublin, CA), and IOP measurement with Goldmann applanation tonometry. Gonioscopy was utilized to confirm that all cases had open angles. Patients were classified as having POAG based on ONH or retinal nerve fiber layer (RNFL) structural abnormalities. The subjects were excluded if they had congenital glaucoma or secondary causes (such as pigment dispersion, pseudoexfoliation, trauma, uveitis, or steroid induced glaucoma) for glaucoma. The control group consisted of patients who attended the ophthalmology clinic for refractive errors, routine ophthalmic examination, or medical staff with no ocular problems. They had no family history of glaucoma and their IOP measurements were <21 mmHg on 2 different visits.

The study protocol was in adherence to the tenets of the Declaration of Helsinki and approved by the Ethics Committee of Hacettepe University School of Medicine. Informed consent was obtained from all study subjects after explanation of the nature and possible consequences of the study.

### Genetic analysis

Venous blood was obtained from the subjects and stored at -20 °C for less than three months before DNA extraction. Genomic DNA was isolated from peripheral blood using the phenol-chloroform extraction method. The genotypes of *APOE, p53*, and *p21* polymorphisms were determined by the PCR-RFLP method.

*APOE* gene polymorphisms were investigated using the primer sequences 5’-GAA CAA CTG ACC CCG GTG GCG-3’ (forward) and 5’-GGA TGG CGC TGA GGC CGC GCT-3’ (reverse). PCR products were checked in 1.5% agarose gel. For RFLP analysis, PCR product was digested with HhaI at 37 °C overnight. Digested DNA fragments were separated on 10% polyacrylamide gel electrophoresis and five different APOE genotypes were observed (*ε3*/*ε3*, *ε2*/*ε3*, *ε3*/*ε4*, *ε2*/*ε4*, and *ε4*/*ε4*).

For *p53* gene polymorphisms, PCR was performed using the primer sequences 5’-CCT GAA AAC AAC GTT CTG GTA A-3’ (forward) and 5’-GCA TTG AAG TCT CAT GGA AG-3’ (reverse) [32]. For RFLP analysis, PCR product was digested with BstUI at 37 °C overnight. Digested DNA fragments were separated on 1.5% agarose gel electrophoresis. Amplified PCR products for *p53* with a C nucleotide at codon 72 (Pro residue) remained undigested and were 448 bp or 432 bp in length depending on whether the 16 bp insertion was present or not. Products with a G nucleotide at codon 72 (Arg residue) were 248 or 232 bp in length depending whether the 16 bp insertion was present or not.

For *p21* gene polymorphisms, PCR was performed using the primer sequences 5’-GTC AGA ACC GGC TGG GGA TG-3’ (forward) and 5’-CTC CTC CCA ACT CAT CCC GG-3’ (reverse) [37]. For RFLP analysis, PCR product was digested with BlpI at 37 °C overnight. Digested DNA fragments were separated on 3% agarose gel electrophoresis. The Ser allele has a single BlpI restriction site (GCTNAGC), resulting in two fragments of 89 bp and 183 bp and the Arg allele remains undigested, producing a single band of 272 bp.

### Statistical analysis

Genotypic distributions were examined for significant deviation from the Hardy-Weinberg equilibrium by a goodness of fit χ^2^ test. The frequencies of genotypes and alleles were compared among glaucoma and healthy subjects using χ^2^ test, whereas non-parametric Kruskal-Wallis and Mann-Whitney U tests were used to evaluate the differences in maximum IOP, cup/disc ratio (C/D), MD and PSD values among *APOE*, *p53*, and *p21* genotypes in POAG group and p<0.05 was considered as statistically significant.

## Results

Seventy-five POAG patients (49 women, 26 men) and 119 healthy subjects (67 women, 52 men) were included in the study. The mean ages were 63.8±9.5 and 61.8±10.2 years in POAG and control groups, respectively (p=0.18). In POAG group, the mean±standard deviations (SD) of the IOP, C/D, MD and PSD were 26.8±3.5 mmHg, 0.6±0.2, 9.7±7.7 dB, and 5.2±4 dB, respectively. The distribution of genotypes was given in detail in Table 1**.** The observed genotypes did not show deviation from the Hardy-Weinberg equilibrium in either the cases or the controls (p>0.05). There were no significant differences in the distribution of *APOE*, *p53*, and *p21* genotypes between the healthy subjects and POAG patients (p=0.39, p=0.12, and p=0.28 respectively). For the exon 4 polymorphism, five genotypes including *ε3/ε3*, *ε2/ε3*, *ε3/ε4*, *ε2/ε4*, and *ε4/ε4* were observed in our study subjects. *APOE ε3/ε3* was the most common genotype observed both in the glaucoma and the control groups, whereas genotype *ε2/ε2* was not detected in both groups. The genotypes *ε2/ε4* and *ε4/ε4* were rare both in POAG and control groups. APOE allele frequencies also did not differ among POAG and control groups (p=0.14; Table 1).

**Table 1:** *APOE*, *p53*, and *p21* genotype and allelic frequencies.

**Gene/Polymorphisms**	**Genotypes**	**POAG (n=75)**	**Control (n=119)**	**p value**
*APOE* epsilon genotypes	ε 4/4	1 (1.3%)	2 (1.7%)	p=0.38
	ε 2/4	1 (1.3%)	1 (0.8%)	
	ε 3/4	8 (10.7%)	19 (16%)	
	ε 2/3	12 (16%)	9 (7.6%)	
	ε 3/3	53 (70.7%)	88 (73.9%)	
*P53* codon 72	Arg/Arg	19 (25.53%)	41 (34.5%)	p=0.12
	Arg/Pro	44 (58.7%)	69 (58%)	
	Pro/Pro	12 (16%)	9 (7.6%)	
*P21* codon 31	Ser/Ser	56 (76.7%)	100 (84%)	p=0.25
	Ser/Arg	17 (23.3%)	19 (16%)	
	Arg/Arg	1 (1.4%)	0	
**Gene/Polymorphisms**	**Alleles**	**POAG (n=75)**	**Control (n=119)**	**p value**
*APOE* epsilon alleles	ε2	13 (8.7%)	10 (4.2%)	p=0.14
	ε3	126 (84.0%)	204 (85.7%)	
	ε4	11 (7.3%)	24 (10.1%)	

The observed genotypes did not show deviation from the Hardy-Weinberg equilibrium in either the cases or the controls (p>0.05). There were no significant differences in the distribution of *APOE*, *p53,* and *p21* genotypes between the healthy subjects and POAG patients.

There were no significant differences in maximum IOP, MD, and PSD values among different groups of *p53* and *p21* genotypes (Table 2). For *APOE* polymorphisms, these parameters were similar in *ε2ε3* and *ε3ε3*, with no statistically significant differences, whereas POAG subjects with the *ε2ε3* genotype had worse visual field defects (median PSD=2.2) than those with the *ε3ε4* genotype (median PSD=1.77; p=0.01) and POAG subjects with the *ε3ε3* genotype had worse MD and PSD values (median= -7.4 and 3.4, respectively) than those with the *ε3ε4* genotype (median= -4.1 and 1.77, respectively; p=0.034 and 0.028, respectively). Only one subject had the *ε2ε4* genotype and he had very advanced glaucoma and one subject with early stage glaucoma had the *ε4ε4* genotype.

**Table 2:** Maximum IOP, Cup-Disc Ratio, MD, and PSD values among different groups of *APOE*, *p53*, and *p21* genotypes.

**Gene**	**Genotype**	**MD**	**PSD**	**Cup-disc ratio**	**Maximum IOP (mmHg)**
		**mean±SD (median)**	**mean±SD (median)**	**mean±SD (median)**	**mean±SD (median)**
*APOE*	ε2/3	-9.00±8.00 (-4.72)	5.47±4.51 (2.21)	0.58±0.22 (0.6)	27.36±4.38 (28.00)
	ε2/4	-18.62 (-18.62)	9.29 (9.29)	0.8 (0.8)	28.00 (28.00)
	ε3/3	-10.41±7.85 (-7.41)	5.40±4.06 (3.4)	0.596±0.214 (0.5)	27.01±3.49 (26.00)
	ε3/4	-4.65±3.01 (-4.09)	2.23±1.41 (1.77)	0.51±0.18 (0.5)	24.62±1.99 (24.00)
	ε4/4	-4.29 (-4.29)	2.62 (2.62)	0.3 (0.3)	24.00 (24.00)
	p value	0.09	0.06	0.5	0.1
*P53* codon72	Arg/Arg	-10.13±7.4 (-8.45)	5.33±4.54 (2.45)	0.56±0.21 (0.5)	26.8±3.1 (25.5)
	Arg/Pro	-9.71±8.1 (-7.01)	5.34±4.09 (3.4)	0.60±0.22 (0.65)	26.2±3.2 (25)
	Pro/Pro	-8.9±7.0 (-5.63)	4.11±2.85 (2.12)	0.56±0.21 (0.55)	29±4.5 (28.5)
	p value	0.7	0.9	0.7	0.1
*P21* codon31	Ser/Ser	-9.69±7.17 (-7.28)	5.25±4.16 (2.74)	0.58±0.21 (0.6)	26.91±3.53 (26.00)
	Ser/Arg	-9.13±9.21 (-4.97)	4.58±3.61 (2.31)	0.57±0.20 (0.5)	26.56±3.59 (25.00)
	Arg/Arg	-	-	0.3 (0.3)	22.00 (22.00)
	p value	0.4	0.8	0.9	0.7

Details of clinical features of 75 POAG patients and 119 unaffected controls for each genotype are shown in the table. IOP indicates maximum intraocular pressure; C/D indicates cup-disc ratio of optic nerve; MD and PSD indicate visual field indices and mean±SD (median) indicates standard deviation from the mean. There were no significant differences in maximum IOP, MD and PSD values among different groups of *p53* and *p21* genotypes. POAG subjects with ε2ε3 genotype had worse visual field defects and POAG subjects with ε3ε3 genotypes had worse MD and PSD values.

## Discussion

Glaucoma is a neurodegenerative disease with multiple genes contributing to the pathogenesis, clinical features and response to treatment. Genetic association studies defining susceptibility to POAG may provide important insights into the pathogenesis, but should be treated with caution until the findings are independently replicated.

In this study, we could not show an association between APOE genotypes/alleles and POAG. The most common genotype was *ε3/3* observed in 70.7% of our patients with POAG and 73.9% of the control subjects and the *ε3* allele was observed in 84% of patients with POAG and 85.7% of control subjects, which were not statistically different. The frequency of the *APOE* *ε4* allele in our control and POAG groups were 10.1% and 7.3%, respectively. POAG subjects with *ε2ε3* and *ε3ε3* genotypes had worse visual field results compared to subjects with the *ε3ε4* genotype. Ressiniotis et al. [22], Lake et al. [23], and Zetterberg et al. [24] have shown that the APOE genotype or alleles do not constitute a risk factor for POAG and NTG, comparable with our results. In the study of Ressiniotis et al. [22] in English population, the frequency of the *ε3* allele was 72.6% in POAG group and 76% in control group and the frequency of the *APOE* *ε4* allele in their control population was 13.3%, which was not different than the glaucoma group (14.6%). In their study, Lake et al. [23] found no significant difference in frequency of *APOE* *ε3* and *ε4* alleles between the normal tension glaucoma group (73.9% and 17.1%, respectively) and the control population (76.5% and 15.5%, respectively). In addition, comparing those patients with progressive NTG disease to the controls revealed no association between APOE genotype and the disease progression. In the study of Jia et al. [25], *ε2*, *ε3*, and *ε4* frequencies were found to be 8.75%, 82.25% and 9%, respectively, in Northern Chinese, which were not statistically different between POAG patients and control group. In contrast to these studies, Junemann et al. [17] have shown a significant association between the level of IOP and the *APOE ε2* allele in German patients, and Vickers et al. [14] showed that the *APOE ε4* allele was associated with elevated risk for NTG in the Tasmanian population. In a recent study [18], the frequency of the *APOE ε4* allele in POAG group was significantly higher, whereas the frequency of the *APOE ε2* allele was found to be significantly lower than those in control group in Chinese population. In contrary, Mabuchi et al. [19] found a significantly lower frequency of the *APOE ε2* and *ε4* alleles in Japanese patients with OAG, and Lam et al. [20] found lower frequency of the *ε4* allele in patients with NTG, but not with high tension glaucoma in Chinese, indicating a protective effect of the *ε4* allele against glaucoma. In a study by Fan et al. [21], *APOE* *ε4* carriers were found to have a decreased NTG risk (p=0.007).

As shown above, there is no consensus whether *APOE* alleles constitute a risk factor or are protective against glaucoma. There are several possible explanations for these discrepancies [19]. *APOE* might have a more obvious effect in populations exposed to different environmental factors or with a different genetic background. The prevalence of NTG, which has a different pathogenesis than POAG is much higher in the Japanese population compared to others. In a previous study, the *APOE* -219G and -491T have been shown to affect optic nerve damage and visual field loss in glaucoma, which supports the importance of *APOE* expression and interaction with *MYOC* polymorphisms in the disease pathogenesis [38]. However, this association was not confirmed in the following studies done in Northern China [25], Southern China [20,21], and in England [39].

Previously, a specific functional polymorphism in exon 4 of *p53* (Arg72Pro) was shown to alter its ability to induce apoptosis in vitro, with the Arg72 variant having enhanced apoptotic potential [40]. As the death of RGC in glaucoma has been proved to be way of apoptosis, many investigators examined whether common sequence variations in *p53* are associated with POAG [26,27,41]. In two separate populations, different polymorphic variants in *p53*, codon 72, were shown to be associated with POAG, the arginine form being a risk factor in the British population [32], and the proline form in Chinese population with an odds ratio of 2.4 [31]. In this study, we did not observe any significant difference in the distribution of *p53* codon 72 polymorphism between the control group and POAG patients, which is consistent with the results of Acharya et al. [42] and Dimasi et al. [43]. In the Indian population, the genotype distribution was 26.8%, 50.9%, and 22.3% for Arg homozygote, Pro/Arg heterozygote and Pro homozygote, respectively, whereas 34.3%, 44.8% and 20.9% in the glaucoma group [42]. In the white Australian cohort, Dimasi et al. [43] found that the *p53* codon 72 Arg/Pro polymorphism was associated neither with the development of glaucoma (high or normal tension) nor with phenotype characteristics like age of onset or severity of glaucoma.

DNA insult can cause activation of *p21*, either directly or through transactivation by wild type *p53.* Cancer research has revealed that mutations of *p21* are very rare and that SNPs are more likely to have a functional effect. The distribution of the *p21* codon 31 polymorphism differs among different ethnic groups with a frequency of the Arg allele ranging from 4% in the white population [35] to 50% in Chinese people [44]. Tsai et al. [36] demonstrated that the Arg allele of the *p21* codon 31 polymorphism was more frequent in POAG patients (56%) compared to healthy individuals (36%) in the Chinese population. In our study, we could not find any association between *p21* codon 31 polymorphism and POAG, consistent with the study of Ressiniotis et al [37]. Arg allele was found in 8% of our control group and 13% in the POAG group. In the study of Ressiniotis et al [37] the distribution of the genotypes in the control subjects was 61 (83.6%) Ser homozygotes and 12 (16.4%) Ser/Arg heterozygotes, which was exactly the same with our control group.

The pathogenesis and genetic risk factors for glaucoma are not fully understood yet. Genetic polymorphisms in *APOE*, *p53*, and *p21* have been investigated in several studies in different populations. Polymorphisms have important implications in human genetic studies and screening for such alleles helps in the detection of a genetic predisposition to disease. However, there are conflicting results about the association of these polymorphisms with glaucoma development and phenotype. The main problem in identifying the gene variants associated with susceptibility to common diseases is that the observed results are not replicated in subsequent studies that used different populations and/or larger numbers of cases versus controls. This discrepancy in the literature may reflect sampling bias, as some of the studies have small number of subjects or it could be attributed to ethnic disparity. Also in glaucoma studies, the inclusion of a normotensive glaucoma group, which has risk factors other than elevated IOP and therefore has a different etiopathogenesis, may make a study more sensitive to underlying neurodegenerative risk factors.

This is the first population study in Turkish POAG patients for multiple polymorphisms that might be associated with POAG. Our study found no link between polymorphisms in *APOE, p53*, and *p21* and POAG in Turkish subjects, although a larger sample is required to clarify the role of these polymorphisms in the pathogenesis and course of glaucoma if their effects are mild.
